# Research Trends in Pain Management After Thoracoscopic Surgery (2015–2024): A Bibliometric Analysis

**DOI:** 10.1155/prm/2700117

**Published:** 2026-03-13

**Authors:** Dezhou Jiang, Funing Liu, Zehao Liu, Jun Peng, Yan Liu, Yan Weng, Run Wang, Qing Zhong

**Affiliations:** ^1^ Department of Anesthesiology, Chengdu Jinniu District People’s Hospital, Chengdu, Sichuan, China; ^2^ Graduate School, Chengdu Medical College, Chengdu, Sichuan, China, cmc.edu.cn; ^3^ Department of Anesthesiology, The People’s Hospital of Jianyang/The Affiliated Jianyang Hospital of Chengdu Medical College, Jianyang, Sichuan, China; ^4^ Department of Anesthesiology, The Affiliated Hospital of Southwest Medical University, Luzhou, Sichuan, China, ahswmu.cn

**Keywords:** analgesia, bibliometric analysis, Bibliometrix, Citespace, enhanced recovery after surgery, pain management, postoperative pain, video-assisted thoracoscopic surgery, VOSviewer

## Abstract

**Background:**

Thoracoscopic surgery, a representative minimally invasive approach in thoracic surgery, is increasingly employed. However, postoperative pain remains a significant barrier to recovery and quality of life. We aimed to quantitatively analyze research on postoperative pain following thoracoscopic surgery over the past decade; to identify the current research landscape, research hotspots, and future trends; and to provide a reference for future studies.

**Methods:**

Publications on postoperative pain following thoracoscopic surgery from 2015 to 2024 were retrieved from the Web of Science database. CiteSpace, VOSviewer, and Bibliometrix were used to analyze publication trends, journals, authors, institutions, countries, regions, and keywords.

**Results:**

The number of publications increased steadily between 2015 and 2024. China and the United States were the leading contributors, forming a global collaboration network with Europe. Major contributing institutions included Tongji Medical College, Guangzhou Medical University, and the Cleveland Clinic. Leading authors included Jianxing He, Hengrui Liang, and Ali Alagoz. Research areas spanned thoracic surgery, pain medicine, and anesthesiology. Frequently cited keywords were “pain,” “rapid recovery,” “analgesia,” “pain management,” “paravertebral nerve block,” and “erector spinae plane block.” Key research themes included multimodal analgesia, chronic pain, and quality of life.

**Conclusion:**

Research on postoperative pain after thoracoscopic surgery has evolved from clinical observation to multimodal analgesia, making advancements toward precision medicine and long‐term outcomes. Current research hotspots include optimizing analgesic strategies, understanding pain mechanisms, refining surgical techniques, and promoting rapid recovery. Promising areas include regional analgesia techniques, liposomal bupivacaine, chronic pain prevention, opioid‐sparing strategies, and spontaneous ventilation anesthesia.

## 1. Introduction

Video‐assisted thoracoscopic surgery (VATS) is a representative minimally invasive technique in thoracic surgery. Owing to its advantages of minimal trauma and rapid recovery, it is widely used in clinical practice [1]. However, postoperative pain remains a key factor hindering rapid recovery and affecting long‐term quality of life. Compared with traditional open thoracotomy, VATS significantly reduces postoperative pain, shortens hospital stays, and lowers medical costs [2]. However, pain management after VATS continues to present significant challenges.

Severe postoperative pain not only increases immediate risks such as hypoxemia, hypercapnia, and elevated myocardial workload [3], but may also lead to postoperative pain syndrome and perioperative neurocognitive dysfunction [4]. More concerning approximately 10%–50% of patients develop chronic pain due to inadequate acute pain control in postoperative settings including VATS [5], leading to impaired physical function and increased psychological burden. Therefore, developing standardized multimodal analgesia strategies that ensure patient comfort, promote early mobility, and reduce complication risk has become a critical component of the Enhanced Recovery After Surgery (ERAS) protocol [6].

In recent years, research on postoperative pain management after VATS has grown rapidly; however, scattered research topics and the lack of systematic reviews have become increasingly evident. Bibliometrics offers a methodological framework for comprehensively assessing the current state of the field by quantitatively analyzing the development trajectory, collaborative networks, and academic influence of scientific literature [7].

In this study, we aimed to analyze data from the past decade using multidimensional bibliometric methods to identify research hotspots, evolutionary pathways, and emerging directions in this field, to provide evidence‐based guidance for clinical practice and research planning.

## 2. Materials and Methods

### 2.1. Data Sources and Search Strategy

Data for this study were obtained from the Web of Science Core Collection database. The search strategy was: TS = (“VATS” OR “video‐assisted thoracic surgery∗” OR “video‐assisted thoracoscopic surgery∗” OR “thoracoscopic surgery”) AND TS = (“pain” OR “postsurgical pain” OR “chronic postsurgical pain” OR “acute postoperative pain” OR “acute postsurgical pain” OR “postoperative pain”), with the time span from January 1, 2015, to December 31, 2024. The inclusion criteria were as follows: (1) studies focusing on postoperative pain after thoracoscopic surgery and (2) articles published in English. The exclusion criteria were as follows: (1) papers that did not align with the study topic and (2) duplicate publications. A total of 1098 papers were retrieved. After excluding 587 non‐topic‐related papers, 511 papers were included in the final analysis. Publication characteristics were obtained from the Web of Science Core Collection database, including publication date, countries and regions, institutional and author collaborations, disciplines, journals, and keywords.

### 2.2. Data Extraction

The search was conducted on May 14, 2025, to capture complete annual data for the year 2024. All searches were performed on the same day to avoid discrepancies due to daily database updates. Screening was performed independently by two reviewers, with a third reviewer making the final decision in cases of disagreement. As this study utilized secondary bibliometric data without personal identifiers, ethical approval and informed consent were not required. The bibliometric data were derived from secondary sources and contained no personal information; therefore, informed consent was not required. Bibliometric information collected included publication year, country, journal, number of citations, authors, discipline, institution, and subject categories.

### 2.3. Data Analysis and Visualization

A standardized keyword‐cleaning protocol was implemented to ensure the analytical rigor of the study. Raw keywords obtained from the Web of Science (WoS) were first processed using the Bibliometrix R package for initial normalization of case, hyphenation, and pluralization. The subsequent step involved merging synonyms and disambiguation terms, by mapping them to canonical equivalents. The Medical Subject Headings (MeSH) thesaurus served as the principal authority for this procedure; for example, the term “video‐assisted thoracoscopic surgery” was selected as the canonical form for “VATS”. For novel techniques not yet incorporated into MeSH, such as the erector spinae plane block (ESPB), standardization was guided by a pre‐established set of rules. The entire automated mapping output was then subjected to manual review by two independent reviewers. A consensus‐based approach was adopted to reconcile differences, with adjudication by a senior researcher when necessary, thereby ensuring the consistency of the final keyword lexicon.

The data were organized into tables or visualized using bibliometric analysis software, including CiteSpace (v6.2.R1), VOSviewer (v1.6.20), and Bibliometrix (R v4.3.3).

CiteSpace is a free Java‐based application that provides dynamic visualizations of the evolution of bibliometric networks over time. In this study, CiteSpace was used to visualize keyword co‐occurrence networks, track the progression of research topics, and identify emerging terms.

VOSviewer is a bibliometric tool that visualizes bibliometric networks using distance‐based mapping techniques. In this study, it was applied to: (1) explore collaboration networks among authors and their affiliated institutions through co‐author analysis; (2) examine relationships among countries and regions through co‐occurrence networks; and (3) visualize citation networks and author co‐occurrence networks across journals. In the generated graphs, nodes represent analytical elements, with node size positively correlated with citation frequency. The thickness and length of the connecting lines indicate the strength of association, with thicker lines and shorter distances representing stronger relationships.

Bibliometrix, an R package designed for quantitative research in scientometrics, was used to summarize publications and citation counts on a global map for bibliometric analysis and to visualize cited literature, authors, and keywords.

The parameters for CiteSpace and VOSviewer were meticulously selected to optimize the clarity and validity of the bibliometric networks. In CiteSpace (v6.2.R1), keyword co‐occurrence analysis was configured with the following settings: a 1‐year time‐slicing (2015–2024), a *g*‐index (*k* = 25) to select significant nodes from each slice, and Pathfinder pruning to highlight the most critical pathways in the merged network. Concurrently, in VOSviewer (v1.6.20), a minimum occurrence threshold of 9 was applied to the 1344 original keywords. This threshold was determined through an iterative process aimed at striking an optimal balance between network comprehensiveness and interpretability, which is a common practice in bibliometric studies. Its application yielded a manageable yet conceptually significant set of 97 keywords for subsequent network analysis, effectively filtering out peripheral terms while preserving the conceptual core and ensuring structural clarity. This empirically derived threshold (9) was also consistent with the theoretical value (*m* ≈ 8.54) estimated based on Price’s law. The resulting keywords were then clustered using the VOS technique to identify thematic groups.

## 3. Results

### 3.1. Analysis of Publication Data

**FIGURE 1:**
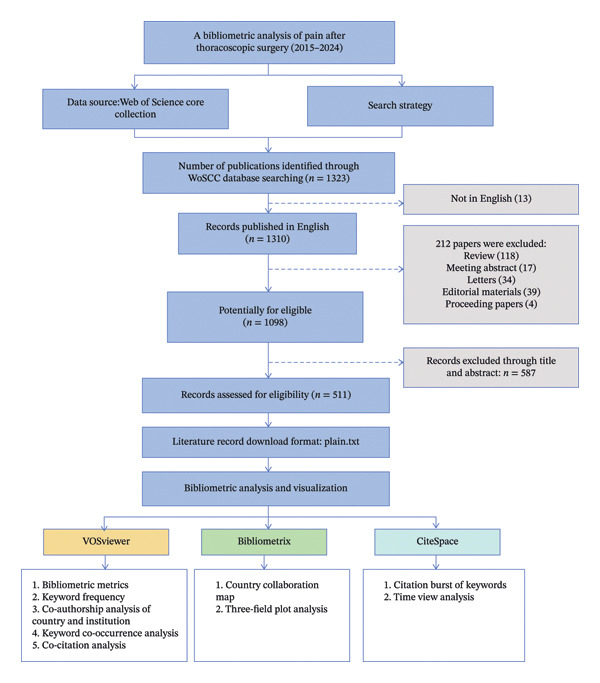
Flowchart of the study.

**FIGURE 2:**
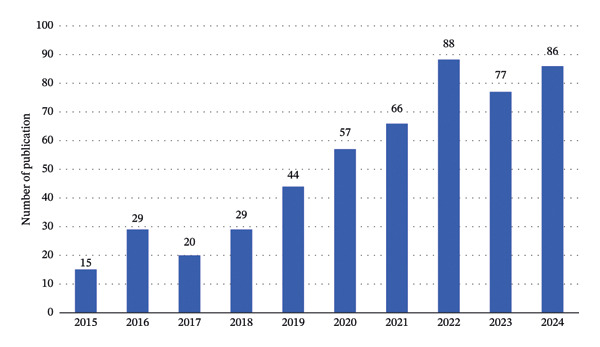
Number of published articles from 2015 to 2024.

An extensive search and screening of the WoS database identified 511 articles (Figure 1). These publications originated from 600 institutions in 37 countries and regions, involved 2808 co‐authors, and were published in 148 journals. They cited 7706 references from 1630 journals. As illustrated in Figure 2, annual publication counts from 2015 to 2024 fluctuated but demonstrated an overall upward trend. A significant increase started in 2019, with output peaking in 2022. The publication peak in 2022 likely reflects multiple converging factors: the maturation of minimally invasive surgical techniques, postpandemic recovery of clinical research, the proliferation of studies on novel regional analgesic methods such as fascial plane blocks, and the stimulatory effect of major international clinical guidelines. These elements collectively accelerated research output during this period.

### 3.2. Analysis of Countries, Regions, and Institutions

Table 1 lists the top 10 countries and regions by publication volume. China ranked first with 241 articles, representing 47.16% of the global total. The United States and South Korea had ranked second and third, with 61 and 41 articles, respectively.

**TABLE 1:** The top 10 countries and regions by publication volume.

Rank	Country/regions	Documents	Citations	Average citations/publication	Percentage of total publications (%)
1	China	241	2582	10.71	47.16
2	USA	61	1382	22.66	11.94
3	South Korea	41	524	12.78	8.02
4	Japan	39	700	17.95	7.63
5	Italy	31	296	9.55	6.07
6	Turkey	26	627	24.11	5.09
7	Taiwan	18	215	11.94	3.52
8	England	15	342	22.8	2.94
9	Germany	12	109	9.08	2.35
10	Denmark	11	950	86.36	2.15

Using VOSviewer, Figure 3(a) visualizes the international collaborative network in post‐thoracoscopic surgery pain research, generated by importing information on leading research countries and regions from the WoS into VOSviewer. The analysis reveals a distinct core‐periphery structure, with China positioned as the central hub, indicating its pivotal role not only in production but also in facilitating global knowledge flow. The thickest connecting lines, particularly between China and the United States, signify the most intensive and likely institutionalized research partnerships, which may be driven by large‐scale bilateral projects or shared clinical trial protocols. Figure 3(b), a density view of the national collaboration map, confirms that China and the United States lead in publication volume. Furthermore, the density map highlights active regional subnetworks, such as intra‐European collaborations and connections within the Asia‐Pacific region, suggesting that geographical proximity and shared regional health priorities also shape the collaborative landscape. Figure 3(c) illustrates the degree of intercountry collaboration, with thicker lines representing stronger partnerships. This visualization underscores the asymmetry in collaboration intensity, where China’s partnerships with European nations (e.g., Germany and England) appear stronger than those with some neighboring Asian countries, potentially reflecting alignment in research infrastructure and funding priorities. Figure 3(d) presents the global distribution of thoracoscopic surgery‐related pain research, graphically underscoring a significant research gap across most of Africa, South America, and parts of Eastern Europe. This stark disparity points to substantial inequities in global research capacity and focus, which should be acknowledged as a limitation and a call for future international outreach.

FIGURE 3(a) Network visualization map of countries and regions related to pain after thoracoscopic surgery. (b) Density visualization of countries and regions contributing to research on pain after thoracoscopic surgery. (c) Map of collaborative networks among all countries and regions. (d) Global distribution of thoracoscopic surgery pain research across countries and regions, displayed on a world map.

**Figure:**
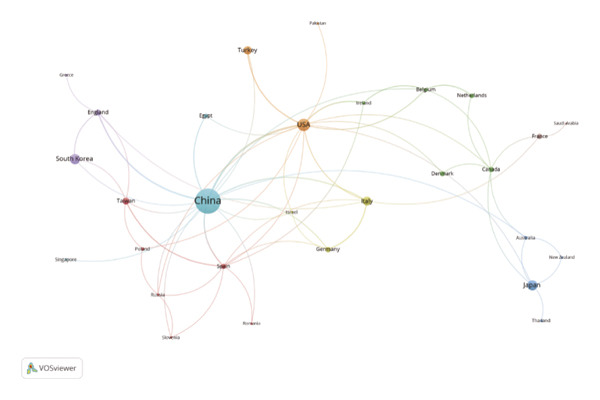
(a)

**Figure:**
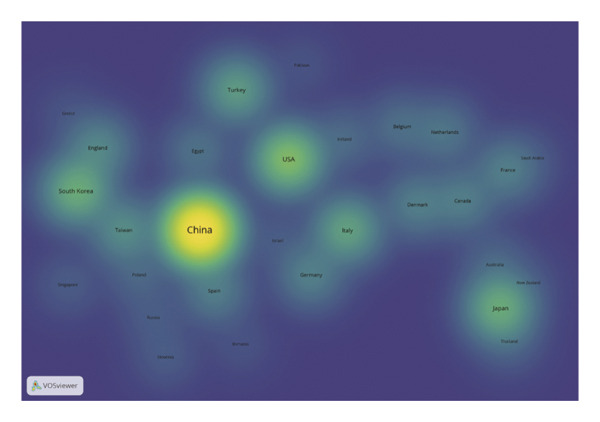
(b)

**Figure:**
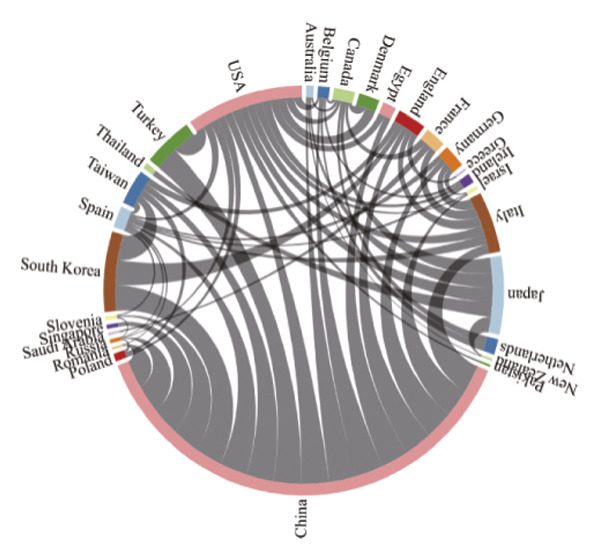
(c)

**Figure:**
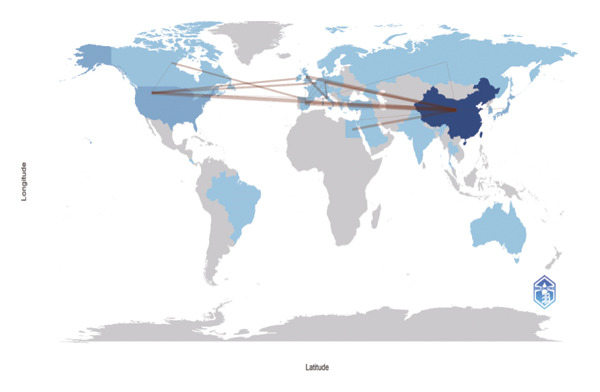
(d)

**FIGURE 4:**
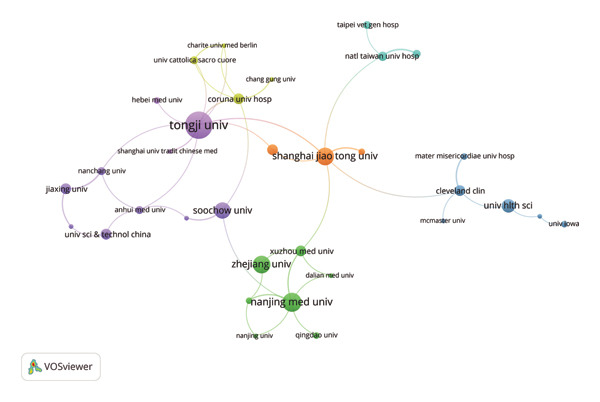
Institution collaboration chart. The dot size represents the number of papers issued by the institution, and different colors represent different clusters.

By importing data on the leading research institutions from the WoS into VOSviewer, the collaboration network among institutions in the field of post‐thoracoscopic surgery pain research is visualized in Figure 4. Table 2 lists the top 10 institutions by publication volume, which are primarily universities and hospitals, with eight being affiliated hospitals of Chinese universities and two being U.S. institutions. The top three by publication count are Tongji University School of Medicine, Guangzhou Medical University, and Nanjing Medical University. The Cleveland Clinic in the United States achieves an average of 29.14 citations per paper, highlighting its strong influence in this research field. Its robust connection with McMaster University forms a critical “evidence‐generation axis,” representing a synergy between high‐volume clinical practice and advanced research methodology. Meanwhile, institutions such as Charité–Berlin University Hospital act as crucial bridges, linking the Chinese clusters with the broader European and North American networks.

**TABLE 2:** Top 10 institutions by publication volume.

Rank	Organization	Documents	Citations	Average citations/publication
1	Tongji University School of Medicine	19	194	10.21
2	Guangzhou Medical University	16	265	16.56
3	Nanjing Medical University	13	70	5.38
4	Shanghai Jiao Tong University School of Medicine	12	107	8.92
5	Zhejiang University School of Medicine	12	163	13.58
6	China Medical University	11	188	17.09
7	Soochow University	11	131	11.91
8	Oregon Health and Science University	9	107	11.89
9	Cleveland Clinic	7	204	29.14
10	University of Science and Technology of China	7	72	10.29

### 3.3. Analysis of Journals

Using VOSviewer, we identified the top 10 journals publishing research on acute and chronic pain following thoracoscopic surgery between 2015 and 2024 (Table 3). These core journals accounted for approximately 40.66% of all publications (209 papers in total). The Journal of Thoracic Disease (53 papers), Journal of Pain Research (28 papers), and Journal of Cardiothoracic and Vascular Anesthesia (26 papers) are the leading outlets in terms of publication volume. Among the top 10 journals, the Journal of Cardiothoracic and Vascular Anesthesia has the highest total citation count, followed by the European Journal of Cardio‐Thoracic Surgery. The citation frequencies of these two journals are markedly higher than those of the other journals.

**TABLE 3:** Top 10 journals by publication volume.

Rank	Source	Documents	Citations	Average citations/publication
1	Journal of Thoracic Disease	53	777	14.66
2	Journal of Pain Research	28	239	8.54
3	Journal of Cardiothoracic and Vascular Anesthesia	26	532	20.46
4	BMC Anesthesiology	17	158	9.29
5	Journal of Clinical Medicine	17	69	4.06
6	Annals of Thoracic Surgery	15	231	15.4
7	European Journal of Cardio‐Thoracic Surgery	14	510	36.43
8	Medicine	14	244	17.43
9	Thoracic Cancer	13	147	11.31
10	Thoracic and cardiovascular surgeon	11	137	12.45

The cited literature spans 1630 journals and covers multiple disciplines, including anesthesiology, surgery, and pain medicine, underscoring the field’s strong interdisciplinary nature.

### 3.4. Analysis of Author Collaboration

According to Price’s law, the number of publications by core authors in a given field is calculated as: *m* = 0.749 × $\sqrt{n_{\max}}$, where *n*_max_ is the number of publications by the most prolific author and *m* is the minimum number of publications required for an author to be considered a core author. In this study, based on VOSviewer statistics, *n*_max_ = 9, resulting in *m* = 0.749 × $\sqrt{9}$ ≈ 2.25.

Therefore, authors with three or more publications were classified as core authors. A total of 148 core authors were identified, collectively publishing 392 papers (accounting for 76% of all publications). These results align with Price’s law and indicate a substantial number of highly productive researchers in this research area.

Table 4 presents the top 10 authors by publication volume. Among the highly productive authors, scholars such as Jianxing He served as key contributors; however, a large‐scale international collaboration network has yet to be established. Jianxing He, ranked first in publication volume, has published nine papers, is affiliated with Guangzhou Medical University, and has received a total of 189 citations, averaging 21 citations per paper. Hengrui Liang, ranking second with seven publications, is affiliated with Guangzhou Medical University in China and has received a total of 68 citations, averaging 9 citations per paper. Ali Alagoz, Musa Zengin, and colleagues, ranked third with seven publications, are affiliated with the University of Health Sciences, Türkiye, and have received 44 citations in total, averaging six citations per paper.

**TABLE 4:** Top 10 authors for publications and citations.

Rank	Author	Documents	Citations	Average citations/publication
1	Jianxing He	9	189	21
2	Hengrui Liang	7	68	9.71
3	Ali Alagoz	7	44	6.29
4	Hilal Sazak	7	44	6.29
5	Musa Zengin	7	44	6.29
6	Lun Liu	6	120	20
7	Ramazan Baldemir	5	34	6.8
8	Gulay Ulger	5	34	6.8
9	Mahmoud Ismail	5	55	11
10	Dania Nachira	5	42	8.4

However, according to VOSviewer statistics, the top three authors by average citation count are Claus Andersen (four publications; average 199 citations per paper) from the University of Southern Denmark, Center for Health Economics Research (COHERE); Emine Ozgur Bayman (three publications; average 76 citations per paper); and Timothy J. Brennan (four publications; average 75 citations per paper).

### 3.5. Analysis of Co‐Citation

Co‐citation analysis identifies frequently cited papers and the journals in which they appear within a specific research field. Using VOSviewer, we generated a co‐citation map of journals, applying a minimum threshold of 30 citations, which yielded 65 journals for analysis. The resulting co‐citation map is presented in Figure 5(a).

FIGURE 5(a) Co‐citation of cited journals; node size represents the co‐citation frequency of the cited journals, and different colors denote distinct clusters. (b) Author co‐citation; node size represents co‐citation frequency, and different colors indicate distinct clusters. (c) Document co‐citation; node size represents citation counts, whereas different colors indicate distinct clusters. (d) Three‐field plot (cited references‐authors‐descriptor/keywords) for pain after thoracoscopic surgery.

**Figure:**
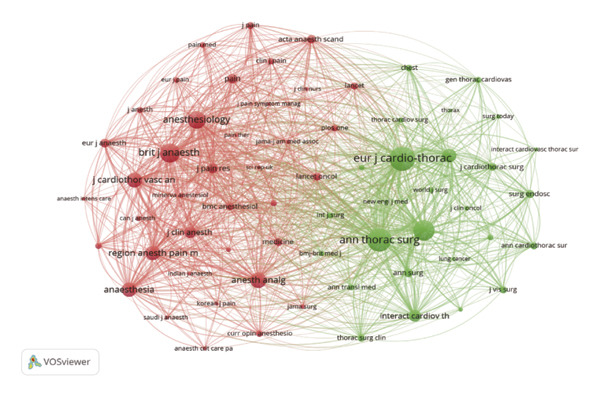
(a)

**Figure:**
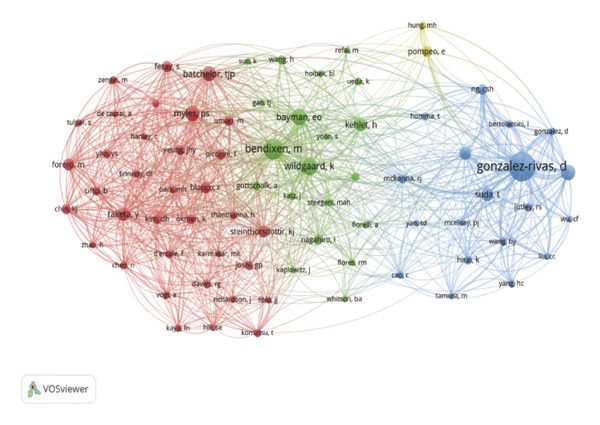
(b)

**Figure:**
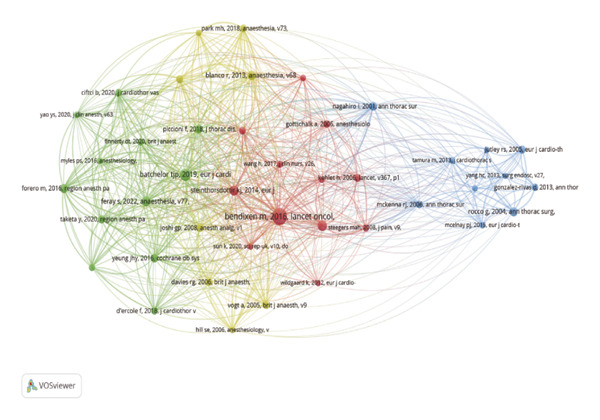
(c)

**Figure:**
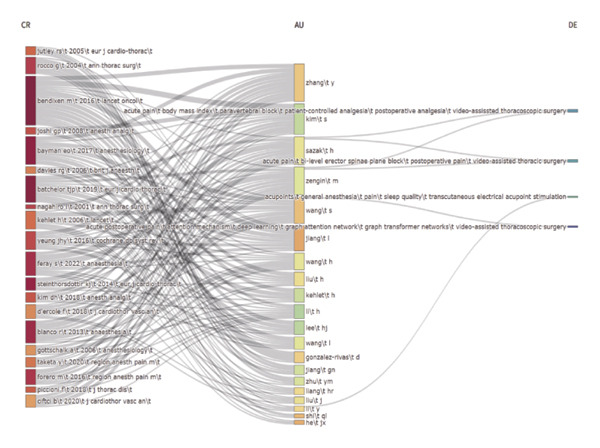
(d)

Figure 5(a) shows that the cited journals form two major clusters: a surgery‐dominated cluster (green nodes) and an anesthesia/pain cluster (red nodes). Annals of Thoracic Surgery and European Journal of Cardio‐Thoracic Surgery, as leading journals in thoracoscopic surgery, primarily cite literature focused on surgical topics such as VATS procedure improvements and post‐thoracoscopic surgery pain research. Within the anesthesia/pain cluster, journals including Anesthesiology, Anesthesia, Pain, British Journal of Anesthesia, Anesthesia and Analgesia, and Regional Anesthesia and Pain Medicine are frequently cited and are considered high‐quality journals in anesthesia and pain management. The robust presence of and strong connections between these high‐impact journals in both clusters confirm that the field’s knowledge base is built upon a solid foundation of rigorous clinical research from both core disciplines. Further co‐citation analysis of authors was conducted using VOSviewer to generate a co‐citation network diagram for the period 2015–2024 (Figure 5(b)). In the diagram, each node represents an author, with node size corresponding to co‐citation frequency. Lines between nodes represent co‐citation relationships, with line thickness indicating co‐citation strength. Color‐coded clusters highlight distinct research themes: the blue cluster is primarily associated with studies on surgical techniques; the green cluster focuses on pain mechanisms and analgesia; and the red cluster encompasses research in perioperative management. The relatively clear separation between these clusters suggests that, although interrelated, these themes have distinct scholarly communities. However, the presence of bridging authors who connect these clusters indicates areas of fruitful integration, such as surgeons investigating enhanced recovery protocols or anesthesiologists developing procedure‐specific analgesic techniques.

Next, VOSviewer was used to generate the co‐citation network of literature references, applying a minimum co‐citation threshold of 20 citations. This resulted in the selection of 42 papers for analysis, with the final co‐citation network presented in Figure 5(c). During the study period, the top 10 co‐cited papers, listed in Table 5, included one guideline, four reviews, and five clinical studies. This composition highlights the field’s evidence‐based approach, valuing primary clinical evidence (clinical studies) and synthesized knowledge (reviews and guidelines). The high co‐citation count of the clinical guideline [6] signifies a major step toward standardizing practice, whereas seminal clinical trials (e.g., [24]) serve as critical benchmarks for establishing the benefits of VATS.

**TABLE 5:** Top 10 co‐cited references related to video‐assisted thoracoscopic surgery postsurgical pain, 2015–2024.

Rank	Citations counts	Author	Year	Title	Journal
1	122	Bendixen M	2016	Postoperative pain and quality of life after lobectomy via video‐assisted thoracoscopic surgery or anterolateral thoracotomy for early‐stage lung cancer: a randomized controlled trial	Lancet oncol
2	65	Batchelor TJP	2019	Guidelines for enhanced recovery after lung surgery: recommendations of the enhanced recovery after surgery (ERAS(R)) society and the European society of thoracic surgeons (ESTS)	Eur j cardiothorac surg
3	52	Bayman EO	2017	A prospective study of chronic pain after thoracic surgery	Anesthesiology
4	48	Steintorsdottir KJ	2014	Regional analgesia for video‐assisted thoracic surgery: a systematic review	Eur j cardiothorac surg
5	43	Blanco R	2013	Serratus plane block: a novel ultrasound‐guided thoracic wall nerve block	Anesthesia
6	43	Feray S	2022	PROSPECT guidelines for video‐assisted thoracoscopic surgery: a systematic review and procedure‐specific postoperative pain management recommendations	Anesthesia
7	39	Forero M	2016	The erector spinae plane block: a novel analgesic technique in thoracic neuropathic pain	Reg anesth pain med
8	37	Yeung JHY	2016	Paravertebral block versus thoracic epidural for patients undergoing thoracotomy	Cochrane database syst rev
9	33	Kim DH	2018	Efficacy of ultrasound‐guided serratus plane block on postoperative quality of recovery and analgesia after video‐assisted thoracic surgery: a randomized, triple‐blind, placebo‐controlled study	Anesth analg
10	27	Park MH	2018	A randomized trial of serratus anterior plane block for analgesia after thoracoscopic surgery	Anesth analg

By importing the screened literature data into Bibliometrix, a three‐field diagram of “literature‐author‐topic” was generated, as shown in Figure 5(d). In this diagram, CR represents the cited literature, AU denotes productive authors, and DE indicates research topic‐related terms. This visualization illustrates the three‐dimensional knowledge network of “literature‐author‐topic” in the field of perioperative pain management in thoracic surgery through three‐field co‐occurrence analysis. For instance, one can trace how a key paper (CR) on ESPB is primarily associated with specific prolific authors (AU) and strongly linked to keywords such as “regional analgesia” and “postoperative pain” (DE), effectively mapping the flow of knowledge from a specific innovation to the researchers and research topics it influences.

**FIGURE 6:**
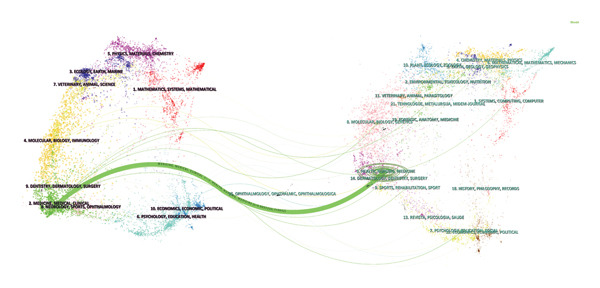
Journal dual‐map overlay of pain after thoracoscopic surgery.

Using Citespace software’s dual‐map overlay technology, the spatial alignment of the literature co‐citation network and the author keyword co‐occurrence network was achieved, as shown in Figure 6. This dual‐map overlay reveals two dynamic mechanisms in thoracoscopic pain research: the horizontal expansion of interdisciplinary collaboration (surgery‐anesthesiology‐neuroscience), and the need for future interdisciplinary collaboration to promote network integration.

### 3.6. Keyword Co‐Occurrence Analysis

In bibliometric research, keywords reflect the core content of a paper. Keyword co‐occurrence analysis helps reveal research hotspots and trends within a discipline.

Using VOSviewer, a co‐occurrence network was constructed from the keywords of 511 publications (Figure 7), where node size denotes frequency, line thickness indicates co‐occurrence strength, and colors represent thematic clusters identified via the clustering algorithm. Figure 7(a) shows the results of a cluster analysis of high‐frequency keywords, which are primarily divided into three clusters: green, red, and blue, representing different research directions.

FIGURE 7Contribution of keywords in the field of pain after thoracoscopic surgery research. (a) Keyword co‐occurrence analysis on pain after thoracoscopic surgery research using VOS viewer. (b) Overlay visualization of the keyword co‐occurrence analysis. The purple nodes represent the keywords that appear earlier, whereas the yellow nodes reflect the recently appearing keywords.

**Figure:**
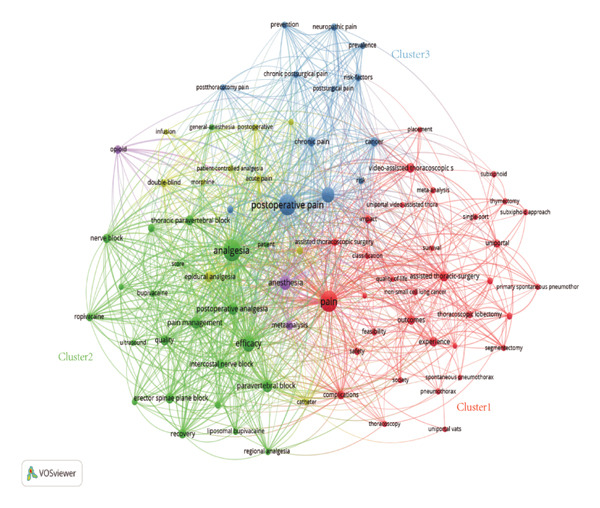
(a)

**Figure:**
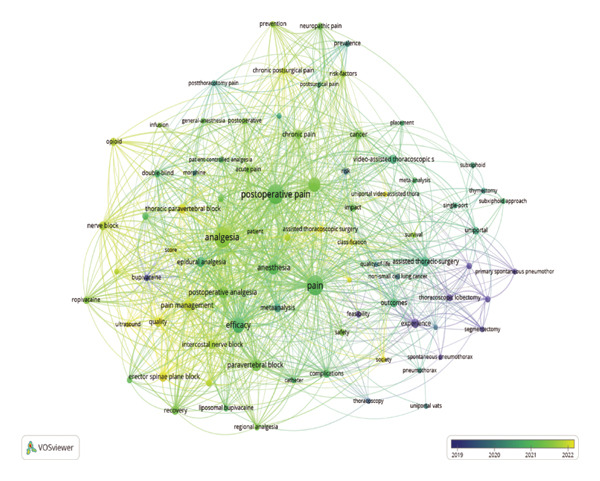
(b)

The keyword co‐occurrence network delineates the field’s intellectual architecture through three interdependent thematic pillars: the Red Cluster, which anchors the research in the surgical foundation and procedural context (e.g., VATS, segmentectomy) linking intervention to pain genesis; the Green Cluster, representing the core analgesic arsenal, where dense interconnections between nerve blocks (e.g., paravertebral block [PVB] and ESPB) and pharmacological agents underscore a focused agenda on optimizing regional analgesia, integrated by the central concept of multimodal analgesia; and the Blue Cluster, which shifts the focus to patient‐centered outcomes and long‐term recovery, emphasizing chronic pain, quality of life, and risk factors. Synthetically, the network reveals a dynamic, goal‐oriented logic: the analgesic cluster serves as the primary interface, addressing the surgically defined pain problem to achieve the outcomes valued in the recovery cluster, marking a paradigm shift toward precision regional anesthesia and a patient‐centric, longitudinal research framework, thus systematically connecting surgical cause, therapeutic intervention, and patient‐centered effect.

The temporal overlay visualization of the keyword co‐occurrence network (Figure 7(b)) reveals the evolutionary trajectory of this research field. Early research foci (blue/purple nodes, 2015–2018) centered on establishing the comparative benefits of VATS over open thoracotomy and on foundational analgesic techniques like epidural analgesia. In contrast, the current research frontier (green/yellow nodes, 2021–2024) is dominated by keywords associated with precision ultrasound‐guided regional blocks (e.g., ESPB and PVB), opioid‐sparing strategies, and the management of chronic pain. This chromatic progression signifies a maturation of the field from validating minimally invasive surgery and conventional pain control toward optimizing patient‐centered, long‐term outcomes through advanced, targeted analgesic techniques.

To quantify the prominence of these themes, the top 20 high‐frequency keywords identified by CiteSpace are listed in Table 6. The strong concordance between the network clusters and this ranked list, where terms such as analgesia, pain, postoperative pain, pain management, PVB, and ESPB dominate, validates the robustness of the identified research structure and confirms that regional anesthesia techniques are at the forefront of current investigative efforts.

**TABLE 6:** Top 20 high‐frequency keywords list.

Rank	Keyword	Occurrences	Total link strength
1	Analgesia	130	707
2	Pain	121	611
3	Postoperative pain	119	629
4	Efficacy	71	451
5	Management	65	382
6	Anesthesia	57	325
7	Paravertebral block	45	261
9	Pain management	39	220
10	Epidural analgesia	32	216
11	Erector spinae plane block	31	178
12	Quality	29	204
13	Assisted thoracic‐surgery	29	158
14	Intercostal nerve block	27	177
15	Chronic pain	27	164
16	Recovery	27	161
17	Experience	27	153
18	Thoracic paravertebral block	27	149
19	Nerve block	25	168
20	Outcomes	25	136

**FIGURE 8:**
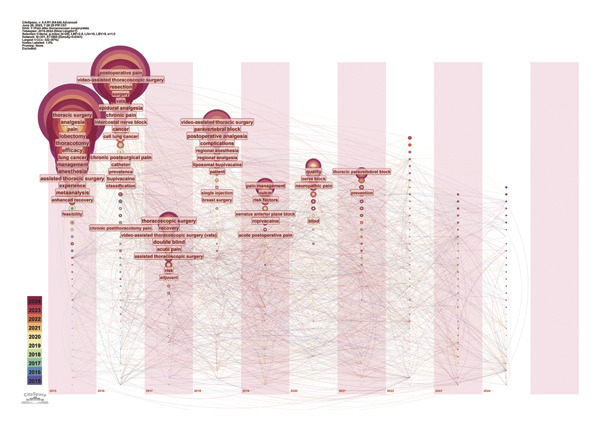
Time‐zone depiction of co‐cited publications on pain after thoracoscopic surgery (2015–2024). The node size represents the frequency of keyword occurrences, whereas the color coding indicates the temporal classification of publication years for each keyword.

Next, we used Citespace software to generate a temporal zone view of keywords (Figure 8), tracking the prominence of key themes from 2015 to 2024. The popularity of each theme over time is indicated by color, whereas the frequency of mentions is visually represented by the size of the circles.

**FIGURE 9:**
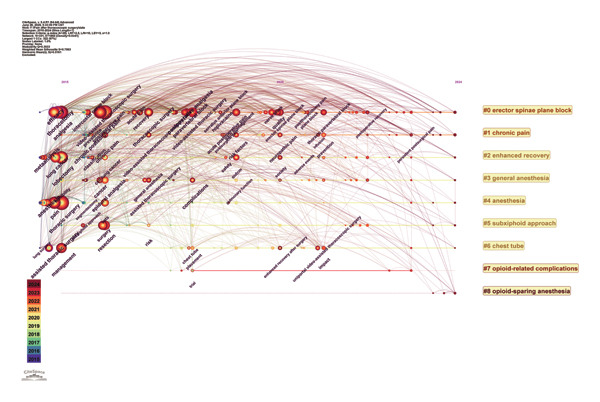
Keyword timeline graph depicting term frequency through proportional node sizing, with chromatic encoding indicating publication‐year clusters.

Subsequently, using Citespace software to plot and cluster keywords via log‐likelihood ratio, we generated the timeline map shown in Figure 9, with a 1‐year time slice (2015–2024). This timeline map highlights the evolution of thoracic surgery research, from technology‐driven to outcome‐oriented, and ultimately to patient‐centered precision medicine. From 2015 to 2018, the focus was on technology‐driven research topics, including “single‐port VATS” and “epidural analgesia.” Between 2019 and 2021, attention shifted toward outcome‐oriented research, with core themes such as “multimodal analgesia,” “ERAS,” and “patient‐reported outcomes.” From 2022 to 2024, precision medicine emerged as a dominant direction, with keywords such as “erector spinae nerve block,” “opioid‐sparing anesthesia (OSA),” “subxiphoid approach,” and “chronic postoperative pain” gaining prominence.

**FIGURE 10:**
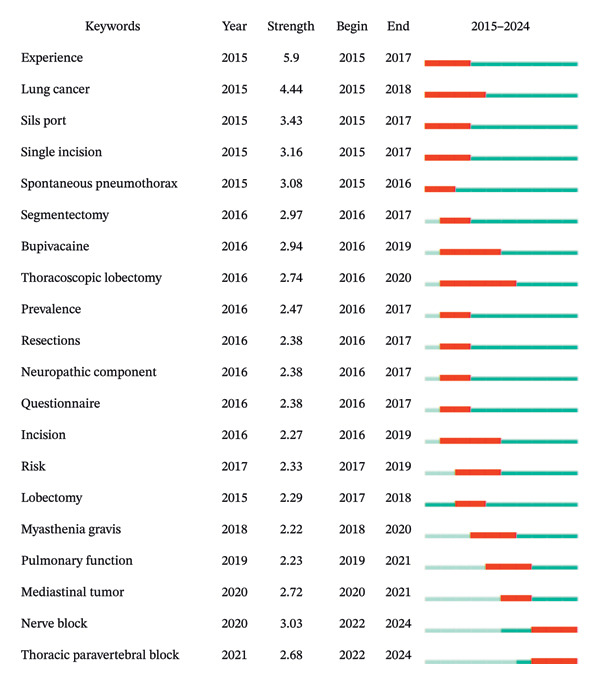
Twenty keywords from publications on pain after thoracoscopic surgery show the strongest bursts of citations. Note: the lengths of red bursts on the blue timelines are proportional to their duration.

Subsequently, a keyword co‐occurrence analysis based on the WoS database was conducted using Citespace covering the time span from 2015 to 2024 (Figure 10). The burst detection method was used to detect keyword bursts, where the length of each bar represents the research intensity (Strength) of the keyword, and different colors denote distinct time periods. Red indicates the burst phase of the keywords, whereas light blue represents the non‐burst phase. The bursts for single‐port thoracoscopic surgery (3.43) and single‐incision techniques (3.16) indicate the initial application of single‐port thoracoscopic technology, which gained widespread attention from 2015 to 2017. The intensity was high; however, the duration was short, reflecting rapid technological iteration. Bupivacaine experienced a sustained burst from 2016 to 2019; as a commonly used regional block agent, this suggests heightened interest in regional block techniques during that period. Since 2022, “nerve block” and “PVB” have emerged as research hotspots, underscoring the demand for combining minimally invasive surgery with precise pain relief and highlighting the role of regional nerve block analgesia as an important component of rapid recovery in thoracic surgery. In contrast, terms related to “chronic postsurgical pain” have not exhibited a burst, possibly due to the longer research cycle and insufficient study scale.

## 4. Comment

In this bibliometric analysis, we collected all publications on post‐thoracoscopic surgery pain research from 2015 to 2024. Subsequently, we visualized and analyzed the information retrieved from the literature through bibliometric software and methods. The analysis revealed the research hotspots and emerging frontiers in post‐thoracoscopic surgery pain research.

### 4.1. Basic Information Analysis

The findings revealed a significant increase in the number of related publications over time, reflecting growing academic interest in this field. This upward trend may be attributed to the integration of ERAS principles into thoracic surgery, the rapid development and widespread adoption of thoracoscopic techniques, and the increasing demand for effective postoperative pain management. Collectively, these factors have shaped thoracoscopic surgery pain management as an emerging area with expanding research directions and clinical value.

The top three countries and regions in terms of publication volume were China, the United States, and South Korea. China ranked first with 241 publications, likely reflecting its large population, the rapid development of minimally invasive thoracic surgery, and increasing research activity in pain management. However, factors such as language barriers, variable research quality, and limited international collaboration have constrained the global influence of Chinese scholars compared with their counterparts from the United States, Denmark, Türkiye, and Spain.

Several research institutions emerged as particularly influential during the study period, such as the Cleveland Clinic, Guangzhou Medical University, and Tongji University School of Medicine. Among these, the Cleveland Clinic stands out as the most influential.

The author collaboration network remains in its formative stage; however, several prominent scholars have already emerged, including a core group of highly productive authors such as Jianxing He. Among relevant journals, the Journal of Thoracic Disease has published the largest number of articles, whereas the Journal of Cardiothoracic and Vascular Anesthesia has achieved the highest citation count.

### 4.2. Research Hotspots and Development Directions in Thoracoscopic Surgery Pain

In bibliometric research, keywords serve as indicators of the core themes and emerging topics within publications. The frequency and evolution of keywords can also reflect trends and dynamics in a given field. Keyword co‐occurrence analysis in this study revealed frequent appearances of terms such as thoracoscopic surgery (VATS), analgesia, pain, postoperative pain, pain management, PVB, ESPB, epidural analgesia, risk factors, bupivacaine, chronic pain, and quality of life, highlighting the main research hotspots during the study period.

Based on these findings, potential future development directions in post‐thoracoscopic pain include: advancements and innovations in surgical approaches; development of new analgesics (such as liposomal bupivacaine); OSA or opioid‐free anesthesia (OFA); nonintubated spontaneous ventilation; refinement of regional block analgesia methods; and expanded studies on chronic pain after thoracoscopic surgery.

#### 4.2.1. Innovations in Thoracoscopic Surgery Techniques

Advancements in thoracoscopic surgery techniques and the exploration of new surgical approaches have driven parallel progress in postoperative pain management. Analysis of the keyword time‐zone view (Figure 8) and timeline chart (Figure 9) provides insights into how research hotspots in thoracoscopic postoperative pain have evolved over the past decade. Postoperative pain following thoracoscopic surgery stems from surgical trauma. Over the past decade, surgical techniques have evolved from open thoracotomy to thoracoscopic surgery [8] and further to single‐port thoracoscopic surgery. Numerous studies have focused on refining surgical techniques to reduce surgical trauma and pain, achieving significant results. Since 2015, the field of thoracoscopic surgery has placed increasing emphasis on surgical experience, with the relationship between technique and postoperative pain remaining a consistent research priority. In terms of surgical techniques, there has been a continuous trend of improvement and evolution from single‐port thoracoscopy to the subxiphoid single‐port thoracoscopic approach. The use of single‐port thoracoscopy (uniportal VATS) in pulmonary resections has expanded rapidly, reflecting the integration and standardization of the ERAS concept in thoracic surgery. Clinical evidence from propensity score matching analyses has shown that the subxiphoid approach for mediastinal tumor resection or lung surgery can significantly reduce postoperative pain [9–11]. These findings underscore the ongoing commitment within thoracic surgery to develop surgical methods that further reduce postoperative pain.

#### 4.2.2. Development of Regional Nerve Block Analgesia Techniques in Postoperative Pain Management for Thoracoscopic Surgery

Regional nerve block analgesia plays a significant role in pain management and anesthesia during thoracoscopic surgery [6]. Over the past decade, it has remained a focus of academic research, with the emergence of novel regional nerve block techniques significantly advancing the ERAS approach. These innovations are expected to remain a priority in future research. Since 2016, intercostal nerve block and epidural analgesia have been widely applied in thoracoscopic surgery pain management techniques.

Subsequently, the field has progressively transitioned toward precision medicine, leading to the development of various nerve block techniques, such as PVB [12], ESPB [13], and serratus anterior plane block (SAPB) [14]. These techniques have demonstrated significant efficacy in managing postoperative pain following thoracoscopic surgery and have become prominent research hotspots. Current evidence recommends PVB or ESPB as the preferred options [15], whereas epidural analgesia is no longer routinely recommended for thoracoscopic procedures [16]. Over the past 5 years, ESPB and uniportal VATS have emerged as the most influential technological milestones in this domain. ESPB has become the new standard for regional analgesia and is closely associated with research on chronic postoperative pain, highlighting the urgent need for improved chronic pain management strategies. Furthermore, studies have confirmed that SAPB is highly effective in controlling postoperative pain following thoracoscopic surgery [17].

Based on bibliometric trends and contemporary evidence, we conclude that while thoracic epidural analgesia remains a highly effective technique, the research focus and clinical preference have shifted toward regional techniques such as PVB and ESPB for VATS within ERAS protocols. These techniques offer comparable analgesic efficacy to epidural analgesia while demonstrating advantages such as fewer side effects and simpler implementation.

#### 4.2.3. Application of New Analgesic Drugs in Pain Management Following Thoracoscopic Surgery

Since 2016, bupivacaine has remained a central focus of research due to its widespread use in early epidural analgesia and PVB. The introduction of the novel analgesic liposomal bupivacaine has further reinforced its prominence in analgesic research [18]. Among these, liposomal bupivacaine represents a significant advance in postoperative pain management, offering the potential for prolonged analgesic formulations.

Recent studies have demonstrated that the novel analgesic liposomal bupivacaine has achieved certain efficacy in postoperative pain management [18]. Furthermore, its combination with SAPB [18], ESPB [19], or intercostal nerve block [20] has been shown to significantly enhance analgesic efficacy and facilitate patient recovery. Previous research has confirmed that the use of liposomal bupivacaine for SAPB or intercostal nerve block during thoracoscopic surgery improves pain control and reduces anesthetic consumption [21].

However, in the burst word analysis, lipid‐based bupivacaine did not display a burst trend, which may be due to the absence of large‐scale studies. In the future, the research and development of long‐acting local analgesics or other novel analgesic drugs will remain a key focus in anesthesia and pain management. Such drugs are applicable to thoracic surgery and have the potential to address analgesic needs across all surgical disciplines.

#### 4.2.4. Research on Chronic Pain Following Thoracoscopic Surgery

In earlier years, research primarily focused on acute pain following thoracoscopic surgery; however, the incidence of chronic postoperative pain remains relatively high [2]. Since 2016, chronic pain following thoracoscopic surgery has garnered attention and become a research hotspot. Beginning in 2019, more studies have examined risk factors for chronic pain, identifying preoperative use of hypnotic medications, surgical techniques, and surgical duration ≥ 2.5 h as contributors to an increased risk of neuropathic pain following thoracic surgery [22]. Studies have confirmed that acute severe pain is associated with smoking history, VATS type, surgical duration, and PCA use; moreover, VATS type, surgery duration, drainage duration, and severe pain on the first postoperative day are independent risk factors for chronic postsurgical pain (CPSP) [23]. Since 2020, the postoperative quality of life after thoracoscopic surgery has gradually gained widespread attention, indicating that perioperative pain management medicine is shifting focus to patient‐centered outcomes and long‐term effects [24, 25]. Future research should prioritize the mechanisms of chronic pain, the clinical translation of novel anesthetic techniques, the long‐term impact of pain on quality of life, and improve evidence quality through multicenter randomized controlled trials.

#### 4.2.5. Advances in Thoracoscopic Surgery Anesthesia

The adoption of nonintubated spontaneous ventilation in minimally invasive thoracoscopic surgery may bring transformative changes to the field. In 2024, OSA and even OFA have garnered significant attention in thoracoscopic surgery anesthesia. The evolution of these approaches has been supported by rapid advancements in regional nerve block techniques, which have also influenced postoperative pain management following thoracoscopic surgery.

In exploring anesthesia techniques for thoracoscopic surgery, OSA and OFA have been increasingly applied in this field, achieving notable results [26]. A study by Qiu et al. demonstrated that, compared with conventional anesthesia, the OSA group had significantly lower maximum pain levels at 6 and 48 h postoperatively [26]. However, because OSA typically incorporates PVB analgesia, the potential influence of nerve block techniques on these outcomes cannot be excluded.

A randomized controlled trial by An et al. [27] found that OFA provides effective analgesia in VATS, achieving the same intraoperative pain threshold index as opioid‐based anesthesia. However, the OFA group exhibited significantly deeper sedation and higher blood glucose levels [27], suggesting that OFA may be less effective than opioid‐based anesthesia in suppressing perioperative stress responses. Other studies comparing postoperative recovery outcomes between OFA and OSA under multimodal analgesia protocols during VATS have reported no significant differences between the two approaches [28].

In studies on OSA and OFA, commonly used medications include dexmedetomidine and lidocaine. However, some studies suggest that lidocaine may not produce a clinically significant reduction in pain scores at 24 h postoperatively [29]. Currently, research findings on OSA are limited, and high‐quality, multicenter studies are lacking, which may become a future research focus.

Non‐intubated spontaneous breathing anesthesia is increasingly being applied in thoracoscopic surgery. Evidence indicates that this technique can reduce postoperative pain after thoracoscopic procedures [30]. Huang et al. reported that, compared with the double‐lumen intubation group, patients receiving non‐intubated thoracoscopic anesthesia experienced reduced postoperative pain, faster recovery, and higher quality‐of‐life scores [31]. Non‐intubation spontaneous ventilation anesthesia has also been safely implemented in thoracic surgery [32]. Since it is often reserved for specific patient populations, its impact on postoperative pain requires further investigation through high‐quality prospective studies.

### 4.3. Limitations

This study has some limitations. First, the analysis was confined to the Web of Science Core Collection, as the CiteSpace software is incompatible with other major databases such as PubMed or the Cochrane Library. Although WoS is a leading source for bibliometric research, this restriction may have resulted in the omission of relevant literature. Second, a potential language and database bias exists due to the exclusive inclusion of English‐language publications, which may overlook significant research published in non‐English journals, such as Chinese core journals. Third, as a purely quantitative method, bibliometric analysis cannot evaluate the methodological quality, risk of bias, or clinical impact of the studies included. For instance, it does not distinguish between randomized controlled trials and observational studies. Fourth, temporal constraints affect the interpretation: the well‐known citation lag means that recently published high‐impact studies may not yet be sufficiently cited to appear prominently in our analysis, and keyword‐based tracking may not fully capture the evolution of terminology for the same technique over time, potentially influencing trend accuracy. Finally, although automated processes were used for data extraction and analysis, supplemented by manual keyword merging, the interpretation of network clusters inherently involves a degree of analytical subjectivity, which delineates the final boundaries of this study’s conclusions.

## 5. Conclusions

In summary, this bibliometric analysis delineates a decade of evolution in post‐thoracoscopic pain research, marked by a paradigm shift from validating surgical techniques toward optimizing patient‐centered outcomes through precision analgesia. Based on the emerging trends identified in our analysis, such as strong citation bursts of nerve block techniques and a growing focus on chronic pain and opioid‐sparing strategies, the following research priorities are proposed to guide future directions in this field.

First, optimization of novel regional analgesic techniques should be prioritized. Although ESPB and PVB are current research hotspots, their comparative effectiveness, ideal dosing regimens (including the role of long‐acting formulations such as liposomal bupivacaine), and impact on long‐term functional recovery remain inadequately explored. High‐quality, multicenter randomized controlled trials are needed to establish standardized protocols.

Second, addressing the challenge of CPSP requires the development of predictive models. The bibliometric data reveal a significant and growing concern regarding CPSP; however, the absence of a keyword burst suggests a gap in high‐impact studies. Future research should therefore prioritize the development and validation of multifactorial prediction models that integrate surgical, anesthetic, psychosocial, and genetic risk factors to enable preemptive and personalized interventions.

Finally, the integration of OSA and OFA into ERAS pathways warrants in‐depth investigation. The recent emergence of OSA/OFA as keywords signifies an important trend; however, their synergy with specific regional nerve blocks and their definitive impact on long‐term postoperative outcomes require further elucidation through well‐designed clinical studies. Such studies should emphasize patient‐centered outcomes, including quality of life, functional recovery, and persistent opioid use.

In this study, we applied visualization analysis to map development trends in this field, providing valuable, up‐to‐date references for future research.

## Author Contributions

Dezhou Jiang: drafting the original manuscript, software, data management, and conceptualization. Funing Liu: formal analysis and conceptualization. Zehao Liu: visualization and software. Jun Peng: visualization and data management. Yan Liu: visualization and data management. Run Wang: visualization and data management. Yan Weng: writing–reviewing and editing, formal analysis, and conceptualization. Qing Zhong: writing–reviewing and editing, supervision, and data management.

## Funding

This study was supported by the Chengdu Medical College 2024 Second Batch of Clinical Scientific Research Fund Projects (Project Number: 24LHTY1‐06), the Jianyang People’s Hospital 2025 Institutional Research Project (JY202569), the Sichuan Provincial Association for the Promotion of International Medical Exchange “Seeking Suitability” Special Research Project (L20230410015), and the Jinniu District Medical Research Project of Chengdu City, Sichuan Province (No: JNKY2024‐37).

## Disclosure

Dezhou Jiang and Funing Liu are co‐first authors.

## Ethics Statement

The authors have nothing to report.

## Consent

The authors have nothing to report.

## Conflicts of Interest

The authors declare no conflicts of interest.

## Data Availability

All data generated or analyzed during this study are included in this article. The dataset generated and analyzed in this study can be obtained from the corresponding author upon reasonable request.
